# Individual differences in intrinsic ankle stiffness and their relationship to body sway and ankle torque

**DOI:** 10.1371/journal.pone.0244993

**Published:** 2021-01-22

**Authors:** Tania E. Sakanaka, Martin Lakie, Raymond F. Reynolds

**Affiliations:** 1 Faculty of Medical Sciences, State University of Campinas, Campinas, São Paulo, Brazil; 2 School of Sport, Exercise & Rehabilitation Sciences, University of Birmingham, Birmingham, United Kingdom; Fondazione Santa Lucia Istituto di Ricovero e Cura a Carattere Scientifico, ITALY

## Abstract

When standing, intrinsic ankle stiffness is smaller when measured using large perturbations, when sway size is large, and when background torque is low. However, there is a large variation in individual intrinsic ankle stiffness. Here we determine if individual variation has consequences for postural control. We examined the relationship between ankle stiffness, ankle torque and body sway across different individuals. Ankle stiffness was estimated in 19 standing participants by measuring torque responses to small, brief perturbations. Perturbation sizes of 0.2 & 0.9 degrees (both lasting 140 ms) measured short- and long-range stiffness respectively, while participants either stood quietly on a fixed platform or were imperceptibly tilted to reduce stability (0.1 Hz sinusoid; 0.2 & 0.4 deg). The spontaneous body sway component (natural random relatively rapid postural adjustments) and background ankle torque were averaged from sections immediately before perturbations. The results show that, first, intrinsic ankle stiffness is positively associated with ankle torque, and that this relationship is stronger for long-range stiffness. Second, intrinsic ankle stiffness is negatively associated with body sway, but, in contrast to the relationship with torque, this relationship is stronger for short-range stiffness. We conclude that high short-range intrinsic ankle stiffness is associated with reduced spontaneous sway, although the causal relationship between these two parameters is unknown. These results suggest that, in normal quiet standing where sway is very small, the most important determinant of intrinsic ankle stiffness may be stillness. In less stable conditions, intrinsic ankle stiffness may be more dependent on ankle torque.

## Introduction

Although posture is flexible and dependent on the coordination of all joints and muscles of the body, it can appear that the single point of the estimated whole body centre-of-mass (COM) is controlled as a means to achieve standing balance [1–5]. Previous studies of unperturbed upright standing have shown that during quiet stance the COM is constantly moving in a pseudo-random manner, producing an unpredictable and irregular sway of approximately 0.5 Hz [6], mostly in the antero-posterior direction [1, 4, 7–10]. Standing is an active, controlled process. Therefore, COM excursions (sway) result mainly from sensorimotor noise of different origins [11–13], although other factors, such as breathing [14, 15], also contribute.

In standing, the ankle joint holds particular interest due to its strategic position linking the long upright body with the horizontal feet, the only body surface normally able to exert gravitational counteractive torques against the ground. Standing demands active neural control of ankle torque because the intrinsic stiffness of the ankle joints is too low for even minimal stability [12, 16, 17]. Nevertheless, by providing an instantaneous torque to counteract gravity or other pulls, intrinsic ankle stiffness can assist the nervous system. Also, by increasing the time constant of the toppling body, i.e. decreasing the rate at which the body accelerates towards the ground when relying only on its intrinsic properties, it allows more time for the nervous system to provide an active response [18, 19].

Intrinsic ankle stiffness has been measured by applying small, brief rotations to the ankle joint and analyzing the resultant torque change. By limiting the time window of the analysis, neural responses are excluded. Previous studies using this approach have revealed several clear tendencies. First, intrinsic stiffness depends on the size of the perturbation used to measure it–it increases as the measuring perturbation becomes smaller [18–21]. Second, intrinsic stiffness is lower if it is measured during or shortly after ankle movement such as body sway [18, 19, 22]. Third, there is increasing evidence that intrinsic ankle stiffness increases as the torque transmitted by the ankle is increased–although conflicting results have been reported [12, 17, 22, 23].

As the main concern in previous studies was to identify the general properties of intrinsic stiffness, differences *between* individuals have not been considered in detail. However a large range of intrinsic ankle stiffness in standing individuals has been reported (from 31% to 135% of gravitational toppling torque) [12, 17–20]. Our aim in the present study was to examine the relationship between an individual’s intrinsic ankle stiffness on the one hand, and their sway velocity and ankle torque on the other. We looked at these relationships when participants were standing normally, and also when we provoked a degree of instability by slowly oscillating the support platform.

## Materials and methods

### Participants

The study was approved by the local human ethics committee at the University of Birmingham (ERN_15–0674). All participants gave a written informed consent. It took place at the School of Sport, Exercise and Rehabilitation Sciences, University of Birmingham, UK. Nineteen healthy adults, aged between 24 and 37 years were recruited for this non-invasive experiment (eight female; age 29.2 ±3.2 years; height 1.71±0.1 m; weight 68.3±11.7 kg; toppling torque per unit angle 10.7±2.3 Nm deg^-1^; mean±SD).

### Procedure and apparatus

**Fig 1:**
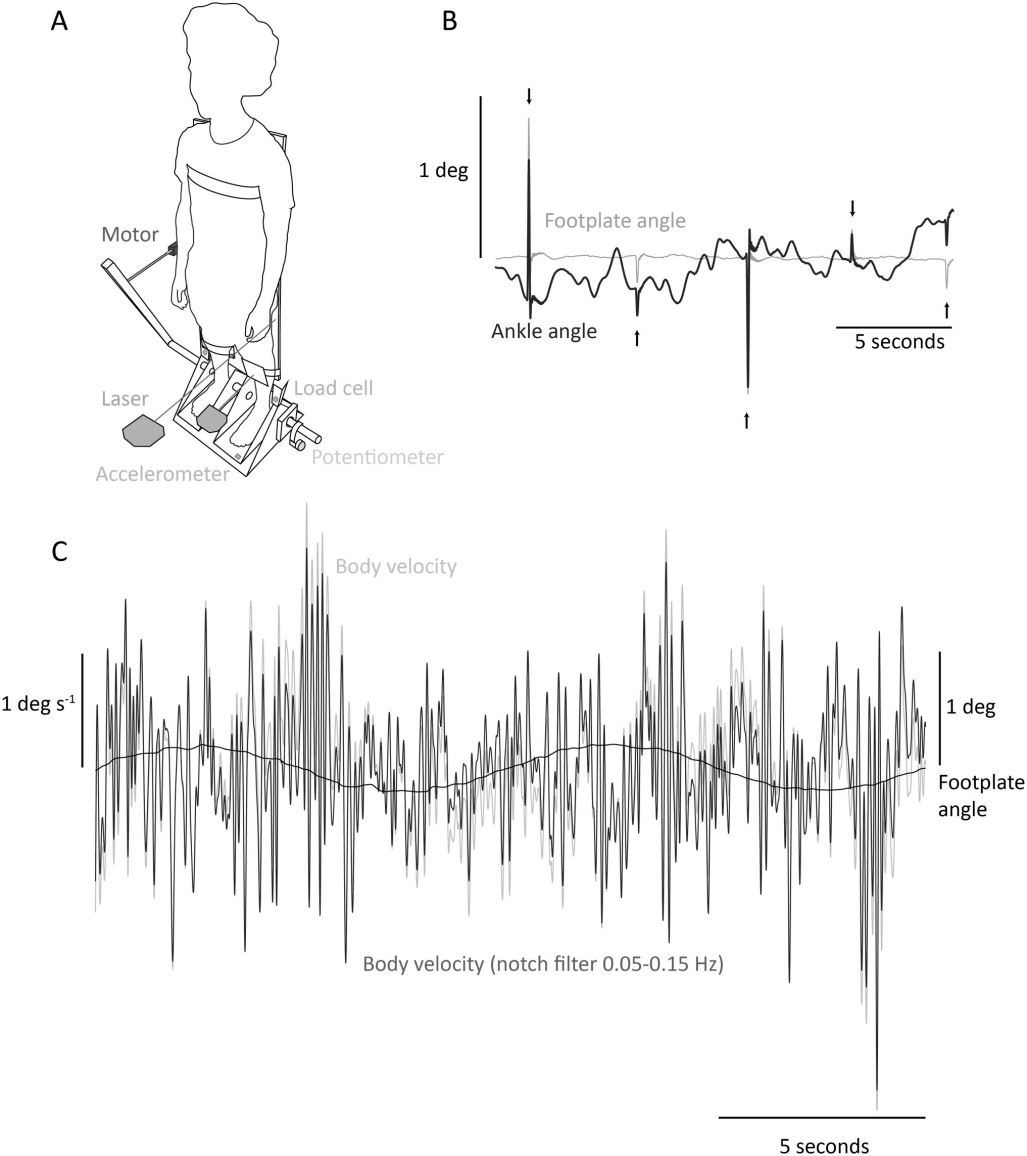
(A) Experimental setup. The servo-motor was installed horizontally and applied perturbations to the crank, thus rotating the platform and footplates. A load cell measured left ankle torque. A potentiometer attached to the axis of rotation measured anteroposterior foot rotation. An accelerometer attached underneath the left footplate measured its acceleration. Two laser-reflex sensors placed at left mid-tibia and at the board tracked the anteroposterior shin and body tilt. Only left lower limb recordings were used for stiffness analysis. (B) Illustrative segment of stiffness-measuring data. Sample data of footplate (gray) and ankle (black) angle during a stiffness-measuring trial, with perturbations indicated by arrows. (C) Illustrative segment of body velocity data. Sample data of body velocity during the sine 0.4 condition. Platform oscillation is shown in black while body velocity is shown in light gray. A notch filter (0.05–0.15 Hz) was also applied to body velocity so that any direct contribution of the slow tilts to body sway was removed (dark gray).

A full description of the footplate apparatus used to measure ankle stiffness, and the associated analysis, is given elsewhere [19] (Fig 1A). In brief, participants were asked to stand on footplates coaxially aligned with their ankles. These footplates were supported by a motorized platform (Copley Motion Systems, Great Britain). Left ankle torque was measured by a miniature load cell (Sensotec Inc., USA) linking the footplate and platform. Left ankle torque, angle, angular velocity and angular acceleration responses to perturbations applied by the platform were recorded for the stiffness estimation. Ankle angle was calculated with a precision Hall effect potentiometer (Midori Precision Instruments Co., Japan) located on the platform axis measuring foot rotation, subtracted from a signal obtained by a laser-reflex sensor (Wenglor, Germany) directed at mid-tibia level and measuring tibia rotation (inverse tangent of linear data from laser-reflex sensor divided by laser height above the ankles). Angular acceleration was measured by an accelerometer located underneath the left footplate (range: ± 3g; Analog Devices Inc., USA). Angular velocity was calculated as the derivative of ankle angle. The methodology specific to the present study is described below.

We were focused on the properties of the ankles, so to reduce the participant’s use of hip and knee strategies a light wooden board (1.2 m length, 0.5 m width and total weight 1.2 kg) was strapped to the participant’s back with Terylene^®^ webbing at shoulder, waist and calf levels. We assumed that, as the board was rigid, its position would give a good estimate of the overall body rotation by minimizing any hip or knee motion. The recording of sagittal body rotation was taken from a laser-reflex sensor (Wenglor, Germany) pointing directly at the board (inverse tangent of linear data from laser-reflex sensor divided by laser height above the ankles), subtracted from foot rotation recorded by the precision Hall effect potentiometer located on the platform axis.

For the measurement of intrinsic ankle stiffness, small, brief raised-cosine type perturbations (140 ms duration) were applied to the footplates. The interval between them (varied between 4 and 5 s, 4.48±0.28 s, mean±SD), and their direction (toes-up or toes-down) and amplitude (0.2 and 0.9 deg), were all randomized. Since we found no difference between toes-up and toes-down estimates of stiffness, these were combined.

Intrinsic stiffness is the resistance to stretch when the ankle is rotated. It becomes greater as the size of the measuring perturbation is reduced so cannot be described by a single value [18–22, 24]. We wanted to measure intrinsic stiffness that would be associated with standing. In normal standing sway, ankle rotations about the medio-lateral axis encompass a wide range of values. A typical mean value for splinted subjects [25] is a little over 0.2 deg with a SD of ~0.1 deg although sway size is very positively skewed. We used two different perturbation sizes (0.2 deg and 0.9 deg) to measure stiffness relevant to small and large sways respectively. For convenience, we refer to intrinsic stiffness measured with smaller and larger perturbations henceforth simply as ‘short-range’ and ‘long-range’.

Each trial period involved 64 perturbations (32 of 0.2 deg and 32 of 0.9 deg). The principle of calculating stiffness by decomposing torque responses to a perturbation is well established [12, 17–20, 23]. In practice, a large number of responses must be averaged together to provide a satisfactory estimate. The reason for this is that, in addition to normal measurement noise, the ankle torque fluctuates due to active changes in muscular force required to balance and is continuously modulated by variation in body angle. Earlier work suggested that a minimum of 25 perturbations were adequate to produce a reliable mean for the condition most influenced by short-range stiffness, normal condition with 0.2 deg perturbations. When measured with more than 25 perturbations, the correlation between actual and estimated torque obtained from the equation described below (1) reached a plateau in which it was consistently similar and close to 1, with an average 0.03% difference from correlation between actual and estimated torque obtained from estimates of averaged 48 perturbations [26]. Therefore, the long- and short-range intrinsic stiffness that we calculate for an individual represents their mean value over a trial (32 perturbations each) and it is these values that are subsequently compared with the mean sway size and mean torque over that trial.

Intrinsic stiffness refers to the natural elastic resistance of the ankle joint to ankle rotation, assuming an unchanging level of muscle activity. It is a passive mechanical property of the muscles and tendons acting at the ankle joint. Passive does not imply that the musculature is relaxed, but that the level of activity is not altered by the nervous system. Conversely, the active mechanism is the alteration of the calf muscle activity by the nervous system.

We examined the relationship between mean intrinsic stiffness, mean sway size and mean torque when the participant stood quietly in a normal attitude on a fixed platform. In separate trials we repeated these investigations when the platform was slowly and slightly sinusoidally tilted (either 0.2 deg or 0.4 deg) about the medio-lateral ankle axis.

We expected that this would induce synchronous sway at the low frequency of the platform and decrease stability. Gurfinkel *et al*. [27] showed that during slow tilting on a platform a prominent feature of signals representing ankle torque and angle was irregular oscillation of small amplitude and relatively high frequency superimposed on the much larger, approximately sinusoidal, body movements. The fluctuations that reflect the normal sway of equilibrium maintenance continue during tilting. We were interested to discover if the size of sway increased during tilting and to see if it was also associated with an individual’s intrinsic stiffness and characteristic torque. Participants were unaware of the experiment protocol and were asked to stand freely with eyes closed to eliminate visual cues.

Therefore, we studied the interrelationship between mean intrinsic ankle stiffness, mean sway size and mean torque in three conditions below:

1. *Normal*: locked footplates;
2. *Sine 0*.*2*: footplates were continuously oscillated by a 0.1 Hz sine waveform of 0.2 deg peak-to-peak amplitude;
3. *Sine 0*.*4*: footplates were continuously oscillated by a 0.1 Hz sine waveform of 0.4 deg peak-to-peak amplitude.

Two trials were conducted for each of the three conditions, totaling 6 trials. Another two trials without perturbations were recorded to normalize data between participants (toppling torque per unit angle assessment, described below). Altogether, 8 trials of approximately 150 s were recorded from each participant. All participants were tested in one session of approximately 2 hours.

### Data analysis

#### Determination of mechanical intrinsic ankle stiffness

The estimation of intrinsic stiffness (K) is described elsewhere [19]. This calculation is based on the assumption that all the structures connected to the ankle joint (the calf muscles, Achilles tendon, aponeurosis and foot) act as a mass-spring-damper system [28, 29] that passively generates the corrective torque applied by the feet against the ground to stabilize position [3, 30]. Torque recorded over the first 70 ms of the perturbation was compared with the torque generated by a second-order fitting equation [12, 24]:

$$T=K\theta +B\dot{\theta }+I\ddot{\theta }$$ (1)

Where: Т = ankle torque (Nm), *θ* = ankle angle (deg), $\dot{\theta }=\text{ankle}\;\text{angular}\;\text{velocity}({\text{deg}\;\text{s}}^{-1})$ and $\ddot{\theta }=\text{ankle}\;\text{angular}\;\text{acceleration}({\text{deg}\;\text{s}}^{-2})$ are the known variables, and K = stiffness (Nm deg^-1^), B = viscosity (Nm s deg^-1^) and I = moment of inertia of the foot (kg m^2^) are the variables estimated from the fitting equation. A 40 Hz low-pass Butterworth filter was applied to the very fast stiffness-measuring data to remove high frequency noise while preserving the integrity of the signal amplitude. The result was the mean intrinsic stiffness of the participant during the trial.

#### Determination of toppling torque per unit angle

There is minimal movement of body parts during normal standing. The range of ankle rotation is normally small enough that gravitational torque exerted by the body COM is very nearly linearly related to the COM rotation around the ankle joint [1–3, 31]. Toppling torque per unit angle is a measure of this relationship and, for small angles, can be referred to as m × g × h (´mgh´), where m is the participant mass above the ankles, g is the gravitational acceleration, and h is the height of the COM above the ankles. As we are interested in between-individual variability, it was important to estimate intrinsic ankle stiffness as a percentage of toppling torque, which effectively normalizes data for height and body mass. However, since the precise height of the COM was unknown (h), we calculated gravitational toppling torque as the slope of the linear fit between ankle torque (load cell data) and body angle (inverse tangent of the laser-reflex sensor data reflecting board rotation divided by laser height above the ankles), recorded during two 150 s trials of voluntary sway, in which subjects were instructed to sway very gently about the ankle joint. Accordingly, we refer to the normalized stiffness values as ‘% mgh’.

#### Determination of normalized ankle torque

In addition to being dependent on the size of the perturbation used to measure it, intrinsic stiffness has been shown to be positively dependent on ankle torque [17, 19, 22, 23]. For this reason, we investigated if the relationship between short- and long-range stiffness *versus* body sway was influenced by baseline ankle torque. We averaged left ankle torque sections of 20 ms prior to each perturbation, after filtering with a 10 Hz low-pass Butterworth filter. This baseline ankle torque was multiplied by 2 to account for two ankles and divided by mgh to normalize it for height and body mass, then multiplied by 100 to obtain % estimates. As ankle torque in standing depends on the size and mass of the subject and on her/his forward angle of lean, normalized ankle torque is a value which is, in effect, the mean angle of forward lean of a participant during the trial.

#### Determination of spontaneous sway

Sway consists of an apparently random fluctuation in body angle and angular velocity during standing. The mean time between stationary instants (points of zero velocity) shown in the literature is around 1 s [32]. As the body sways at a very low frequency, we filtered the recordings of body angle with a 10 Hz low-pass Butterworth filter to reduce the effect of noise [33–35]. With the addition of 0.1 Hz platform oscillations, body sway frequency does not change greatly, so an increase in body angular speed implies an increase in spontaneous sway amplitude. However, to minimize effects of evoked tilt on spontaneous sway estimates, body angular velocity from all 3 conditions was filtered with a notch filter at 0.05–0.15 Hz interval to remove the platform tilt component (Fig 1C). A Savitzky-Golay differentiating filter (polynomial order = 4, window length = 21, sample rate = 1000) was then applied to this signal for the estimation of body angular velocity. This signal represented the angular speed with which the body moved from one temporary stationary instant to the next. Sway was measured as the averaged root-mean-square (RMS) of body angular velocity 1.5 s prior to each perturbation. This value represented the participant’s mean sway velocity during the trial.

### Statistical analysis

Statistical analysis was performed with RStudio: Integrated Development for R (Version 1.1.453, RStudio Team, 2016, RStudio, Inc., Boston, MA, USA). Descriptive statistics (mean and standard deviation) were generated to describe the profile of the sample. Coefficient of variation (CV) was used to determine relative variability between participants in these different conditions. Two-way repeated measures ANOVA was used for each variable to verify if the conditions of increasing platform tilt (normal, sine 0.2 and sine 0.4) and increased perturbation amplitude (0.2 and 0.9 deg) induced significant changes in average intrinsic stiffness, postural sway and baseline torque. Pearson’s correlation coefficient (r) was used to verify the relationship between these 3 variables to answer the main question of this study, i.e. is sway related to stiffness and torque. Following suggestion by Cohen [36], we considered 0.3≤r<0.5 a moderate correlation, and r≥0.5 a strong correlation. P<0.05 was considered statistically significant for all tests.

## Results

### Intrinsic ankle stiffness, baseline ankle torque and spontaneous sway

**Fig 2:**
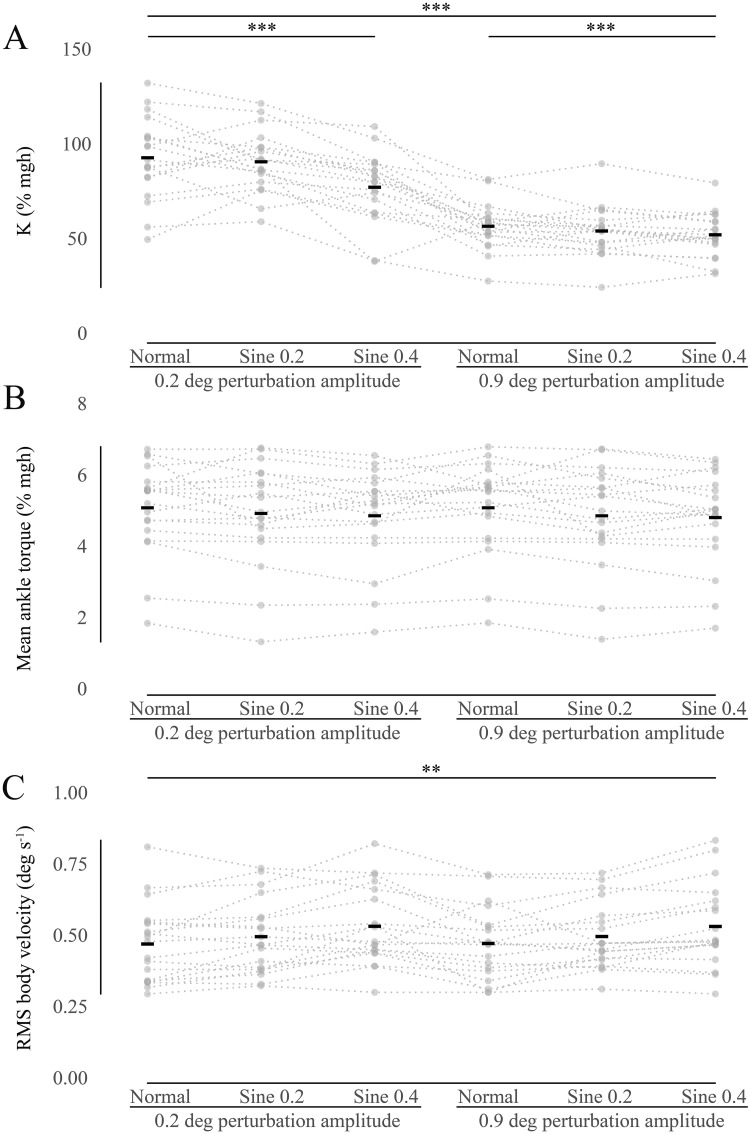
Univariate scatter plot of relevant data. Black rectangles indicate mean values. Dotted lines connect data from each participant. (A) Intrinsic ankle stiffness (K). K estimates during normal, sine 0.2 and sine 0.4 conditions are shown for perturbations of 2 different sizes (0.2 & 0.9 deg). Dotted lines confirm that K reduced in all participants with larger perturbations and increased platform tilt. (B) Mean ankle torque. Same in all conditions. (C) RMS body velocity. Sway progressively increases with increased platform tilts. (**) indicates P<0.01 and (***) indicates P<0.001.

Intrinsic ankle stiffness was measured using both small (0.2 deg) and large (0.9 deg) perturbations, during fixed platform and tilting conditions (Fig 2A). There was a large range of values between individuals, with short-range stiffness (mean±SD = 94.1±21.6, 92.0±16.2, 78.5±18.5% mgh, and CV = 23.0%, 17.6%, 23.5%, in normal, sine 0.2 and sine 0.4 conditions, respectively) being significantly larger than long-range stiffness (mean±SD = 57.9±12.3, 55.4±13.1, 53.4±11.6% mgh, and CV = 21.3%, 23.7%, 21.7%, in normal, sine 0.2 and sine 0.4 conditions, respectively), consistent with previous findings [19]. The main effect of perturbation amplitude was significant, F(1,18) = 201.2, p<.001, as was the main effect of platform tilt, F(1.430,25.735) = 11.9, p <.001, and the interaction of these two factors, F(2,36) = 6.1, p <.010. Mauchly’s Test indicated that the assumption of sphericity had been violated for the main effect of platform tilt, and therefore, a Greenhouse-Geisser correction was used in this case. A post-hoc Bonferroni Test showed that stiffness was significantly lower in sine 0.4 condition when compared with normal and sine 0.2 conditions at the p <.010 level of significance. Stiffness was not significantly different between normal and sine 0.2 conditions.

Estimated viscosity was larger for larger perturbations, F(1,18) = 107.9, p <.001, but remained similar across conditions, F(2,36) = 0.01, p = .988. The interaction of these two factors was significant, F(2,36) = 8.5, p <.010. Increase in viscosity for larger perturbations was expected because 0.9 deg perturbations were much faster than 0.2 deg perturbations, as the raised cosine-type perturbation always had a 140 ms duration. Peak velocity for 0.9 deg perturbations was on average 20.3±0.8 deg s^-1^, and for 0.2 deg perturbations, 5.0±0.4 deg s^-1^. It is a feature of biological tissues such as tendon and muscle that they exhibit non-Newtonian viscosity which is higher at high stretching velocities [37].

Baseline torque did not differ much between conditions (Fig 2B), decreasing only slightly with increasing platform tilt, from normal (5.16±1.28% mgh and 5.16±1.29% mgh, for 0.2 and 0.9 deg perturbation amplitudes, respectively) to sine 0.2 (5.00±1.43% mgh and 4.93±1.40% mgh), and sine 0.4 conditions (4.93±1.33% mgh and 4.88±1.30% mgh). None of the relationships were significant (main effect of perturbation amplitude, F(1,18) = 3.2, p = .092, main effect of platform tilt, F(2,36) = 2.3, p = .119, and the interaction of these two factors, F(2,36) = 1.0, p = .369). As described above, baseline torque is a proxy for mean body attitude which clearly did not change much in the different conditions. It is also evident that, with a few exceptions, most participants seemed to prefer a particular attitude.

Sway, or RMS body velocity of all participants showed a slight progressive increase during the transition from normal (0.48±0.14 deg s^-1^ and 0.48±0.13 deg s^-1^, for 0.2 and 0.9 deg perturbation amplitudes, respectively) to sine 0.2 (0.50±0.13 deg s^-1^ and 0.51±0.12 deg s^-1^), and sine 0.4 conditions (0.54±0.14 deg s^-1^ and 0.54±0.14 deg s^-1^) (Fig 2C). The main effect of platform tilt was significant, F(2,36) = 10.1, p<.001. The other relationships were not significant (main effect of perturbation amplitude, F(1,18) = 0.01, p = .912, and the interaction of these two factors, F(2,36) = 0.02, p = .983). Post-hoc Bonferroni Tests showed that sway was significantly higher in sine 0.4 when compared with normal condition at the p<.010 level of significance and with sine 0.2 condition at the p<.050 level of significance. Sway was not significantly different between normal and sine 0.2 conditions.

### Relationship between intrinsic ankle stiffness, baseline ankle torque and spontaneous sway

**Fig 3:**
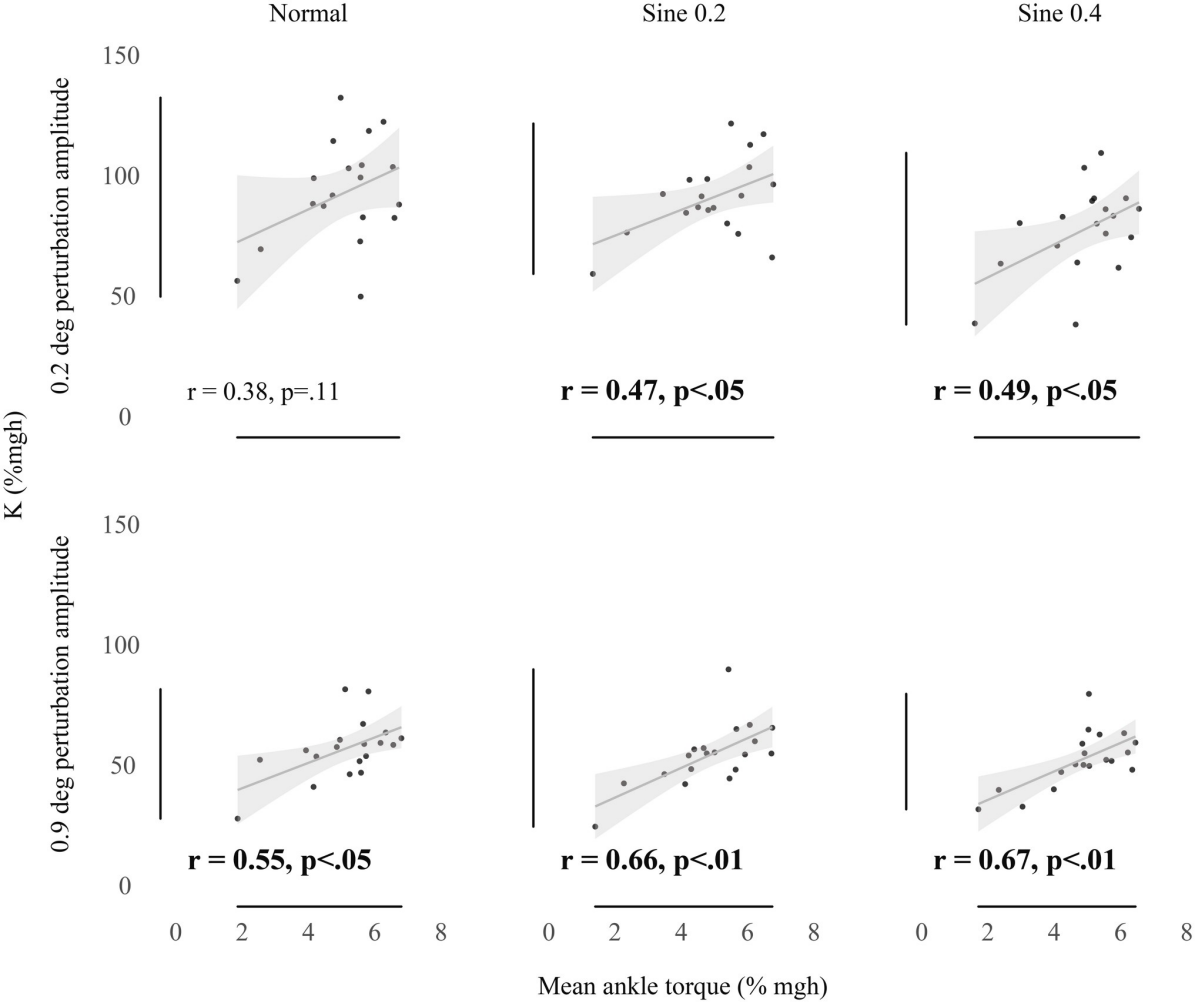
Correlation between K (% mgh) and baseline ankle torque (%mgh). Bivariate scatter plot with regression line and confidence interval band (95% CI) correlating K measured with 0.2 deg (top) and 0.9 deg (bottom) perturbations versus mean ankle torque. Significant correlations are highlighted in bold.

Fig 3 shows that as individuals’ baseline torque increased, intrinsic ankle stiffness tended to increase. This was a strong and significant relationship for all measures of long-range stiffness (0.9 deg perturbation). There was also a positive relationship for all measures of short-range stiffness (0.2 deg perturbation), with significant relationships in sine 0.2 and sine 0.4 conditions.

**Fig 4:**
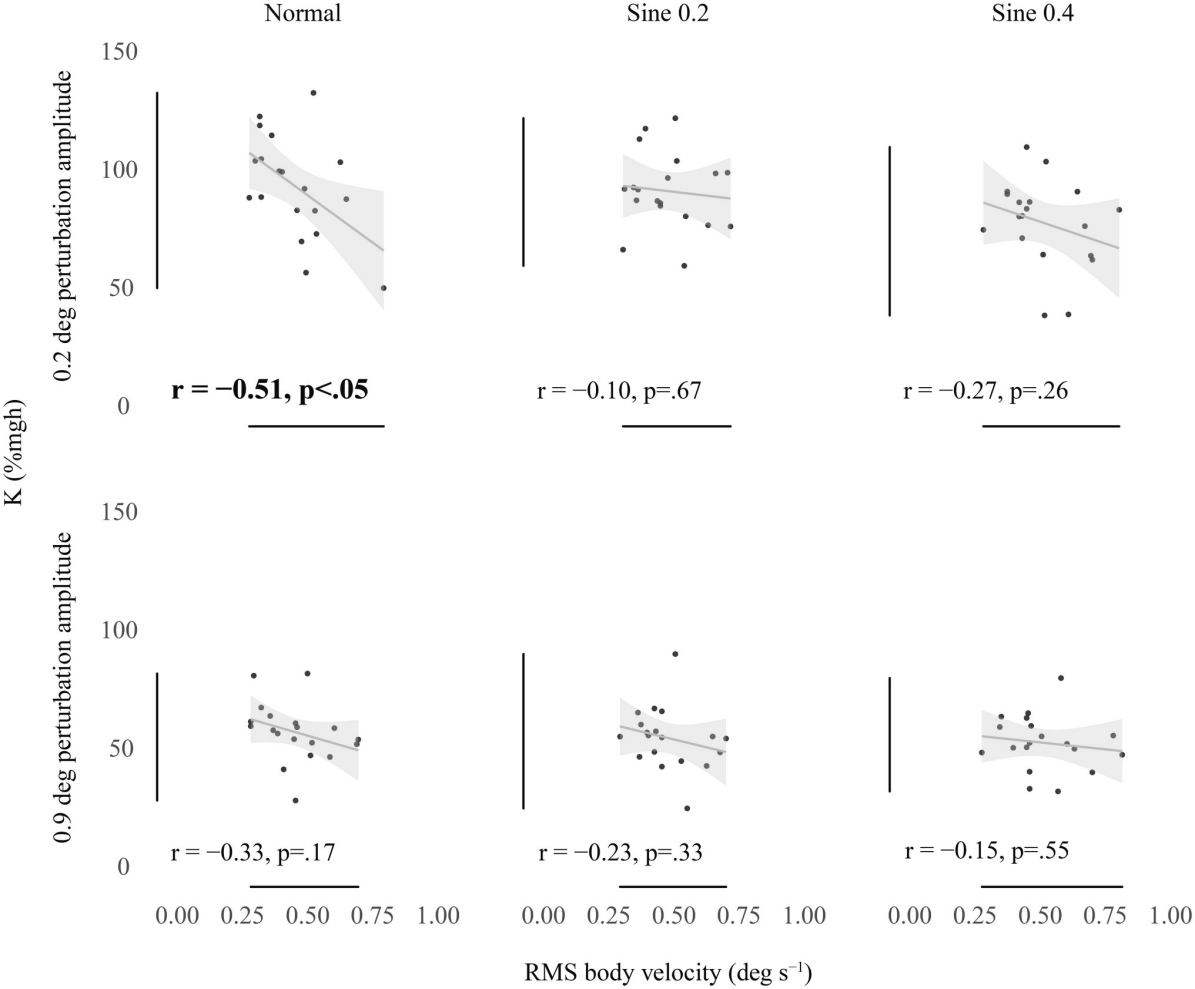
Correlation between K (% mgh) and RMS body velocity (deg s^-1^). Bivariate scatter plot with regression line and confidence interval band (95% CI) correlating K measured with 0.2 deg (top) and 0.9 deg (bottom) perturbations versus average RMS body velocity. Significant correlation is highlighted in bold.

Fig 4 shows that there was a tendency for participants who exhibited higher spontaneous sway to exhibit lower intrinsic ankle stiffness. However, in contrast to the relationship between baseline ankle torque and intrinsic stiffness, this was a weaker negative relationship and only significant for short-range stiffness in the normal condition (0.2 deg perturbation p<0.05).

## Discussion

Previous research has shown that people can have very different intrinsic ankle stiffness [12, 17–20, 22]. Also, previous research has shown that intrinsic ankle stiffness (and other limb stiffness) increases when it is measured with small stretches, and in conditions of minimal movement [38]. Most of these studies show that the intrinsic ankle stiffness has a positive dependency on ankle torque–an exception is the study by Loram and Lakie [12] which measured stiffness with exceptionally small perturbations. Previous research has also shown that people sway by different amounts [33, 39, 40]. Here we examine individual variation in sway, intrinsic ankle stiffness and torque, and we investigate how they are related.

### Range of spontaneous sway velocity

RMS body velocity is a useful measure of postural instability [41, 42] because it reflects the spontaneous sway reversals that naturally occur during standing. Sway was recorded when people were standing normally on a stationary surface. In separate trials, we also slowly oscillated the support surface, causing RMS sway velocity to increase slightly but non-significantly in steps of 0.086–0.093–0.098 deg s^-1^ (normal-sine 0.2-sine 0.4) (Fig 2C). Most participants increased their sway velocity progressively as they were tilted more (Fig 2C). Participants also tilted slowly and in synchrony with the surface, and body tilt usually exceeded the platform tilt, as previously reported [43]. The propensity of participants to tilt, and the relationship to their intrinsic stiffness is the subject of a related investigation (Sakanaka, Lakie, Reynolds unpublished observations). In the present study the slow tilt component was removed by the filtering process that we employed so it did not contribute directly to the spontaneous sway velocity reported here.

### Range of intrinsic stiffness

Our participants were all young healthy adults (29.2±3.2, mean±SD). To our satisfaction, even with this limitation, we found a large range of intrinsic ankle stiffness of 94% mgh difference between maximum (134% mgh) and minimum (40% mgh) values for short-range stiffness and 65% mgh difference (range 91–26% mgh) for long-range stiffness (Fig 2A). When applying very small perturbations (0.055 deg), Loram and Lakie [12] also found a large range of stiffness of 98% mgh (135–37% mgh). Casadio, Morasso and Sanguineti [17] applied larger (1 deg) perturbations, and found a smaller range of stiffness, 33% mgh (81–48% mgh). Amiri and Kearney [22] used a slightly larger perturbation (1.15 deg) and reported a range of stiffness of 41% mgh (14.7% -55.7% mgh). Therefore, the mean values and the range of intrinsic stiffness that we report fit comfortably with previous research which has consistently shown that stiffness is greater with small perturbations [18, 19, 21]. The mean short-range intrinsic stiffness (88.2% mgh) that we measured was higher than long-range intrinsic stiffness (55.6% mgh) (Fig 2A). Amiri and Kearney [22] have recently confirmed that an individual’s intrinsic ankle stiffness is not constant in standing, but can systematically increase with background ankle torque. It is clear that intrinsic ankle stiffness cannot be regarded as constant in a standing, swaying person. However, the present results do suggest that some individuals have, on average, greater stiffness than others. We next examine how their values of intrinsic stiffness might be related to their sway velocity and ankle torque.

### The relationship of individual intrinsic ankle stiffness to spontaneous sway velocity and background torque

We fitted linear regression models between intrinsic stiffness and spontaneous sway velocity (Fig 4). These results show that the strongest correlation and the only significant one occurs for short-range intrinsic stiffness in the normal condition (Fig 4 top left). This condition is one that disturbs the participant to the smallest degree (small measuring perturbations and no tilting). For short-range intrinsic stiffness in sine 0.2 and sine 0.4 conditions and long-range intrinsic stiffness in normal condition the correlation, although seemingly present, is greatly reduced and insignificant (Fig 4 top middle and right and bottom panels). Intrinsic stiffness seems to be related to small sway size only in the condition where the participant is minimally disturbed.

To investigate the relationship between intrinsic stiffness and baseline torque (Fig 3) we also fitted linear regression models. Fig 3 (bottom panels) shows that there is a clear positive relationship between baseline torque and long-range intrinsic stiffness but the same relationship for short-range stiffness (Fig 3 top panels) is weaker and less significant or non-significant. Amiri & Kearney [22] used a different technique to measure intrinsic ankle stiffness (pseudo random binary perturbations). The size of their stimulus was approximately 1 deg which is somewhat larger than our long-range stiffness. They also reported a strong positive relationship between background ankle torque and intrinsic stiffness.

The level of significance chosen in our results (0.05%) is naturally arbitrary, so an economical interpretation of Figs 3 and 4 is that short-range stiffness is most strongly associated with small sway velocity in minimally disturbed conditions, whereas long-range stiffness is most strongly associated with increased background torque. Most sways in standing are extremely small. Loram & Lakie [12] found the median size of ankle rotation in unperturbed standing to be ~0.125 deg. Because intrinsic stiffness depends on the size of the perturbation used to measure it, this raises the question of which size is relevant to standing. Using a technique which decomposes unperturbed standing sway, Zhang *et al*. [44] have shown that passive torque represents over 94% of ankle torque. This result will reflect the fact that in normal standing most sway is very small, so the short-range intrinsic stiffness that we measured in normal condition (mean value 94.1% mgh) is most pertinent to standing. Amiri & Kearney [22] have recently suggested that the increase in longer range (~ 1 deg) intrinsic stiffness with torque may be functionally useful because it will tend to increase intrinsic stiffness as the limit of the standing range is approached. The present results support this suggestion. They also suggest that for shorter range stiffness (~0.3 deg), which is probably rather more relevant to quiet standing, intrinsic stiffness depends less significantly on torque but much more on lack of movement. This is a functional relationship which can work over the whole range of normal standing orientations. Loram & Lakie [12] showed that mean short-range ankle stiffness (~0.05 deg) in standing was approximately 10% higher than it was in the same participants performing an equivalent task (balancing a body sized inverted pendulum with the feet). In this task sway was larger than in normal standing but all other parameters (torque, angle and EMG) were the same. Thus, while in conditions of loss of balance the increase in intrinsic ankle stiffness with torque may be a help, normally the more important parameter may be to minimize movement in order to maintain high short-range stiffness.

### A limitation of the perturbation technique

The data we present (Figs 2–4) describe differences between participants. At an early stage, we wondered if the tendencies that we saw across the group of participants (positive association of their mean intrinsic ankle stiffness with ankle torque and negative association with movement) would be detectible using our technique in individual subjects. Consequently, we looked at the associations between intrinsic stiffness, torque, and sway velocity on a perturbation by perturbation basis for each participant in every trial (data not reported). Although the results did reflect the relationships that we described between individuals, the correlations were generally weak. Intrinsic ankle stiffness and ankle torque did show significant positive correlations in many individuals, particularly for the larger perturbation. Also, there was always a negative correlation between intrinsic ankle stiffness and body sway, but this was nearly always below the level of significance. For a single perturbation, the reactive torque represents only a tiny proportion of the torque that is recorded, so perturbations need to be repeated many times to reduce the biological and instrumental noise. Many such estimates need to be pooled in order to obtain a meaningful value and if correlations are to be made many perturbations are required. Our technique was inadequate to show tendencies in individual trials with the number of perturbations that we used, so we restricted our analysis to pooled individual data. The small perturbations we employed do have the advantage that they minimally disturb the normal standing process, but as they occur on average only every 4.48 s, long periods of standing are needed to provide reasonable estimates of stiffness. Other techniques such as PRBS [22, 24, 29] can provide a more rapid and robust assessment although they may cause more disturbance.

### A possible explanation and questions for future research

These results show that individuals with small spontaneous sway velocity tend to have high short-range intrinsic stiffness. Torque has a smaller and less significant effect on short-range intrinsic stiffness. A possible explanation is that the short-range stiffness of the ankle is limited by compliance of connective tissues. Numerous in vitro and in vivo experiments have shown the existence of high short-range stiffness in skeletal muscle tissue. That is, as a pull is applied to a muscle tendon unit, the muscle length changes very little and the series component extends. Somewhat counter-intuitively, at relatively small tensions, the muscle is much stiffer than the tissues which transmit the forces to the load. A muscle tendon unit cannot be stiffer than its most compliant part so the intrinsic stiffness will reflect the stiffness of the connective tissue. This explains why increasing torque (which will stiffen the muscle by increasing the number of cross-bridges) cannot greatly increase short-range ankle stiffness. Even if the muscle becomes completely rigid the stiffness cannot rise above the limiting value of the series component. (However, because tendon has a non-linear stiffness there will be a small increase in intrinsic stiffness with increased tension which is the general tendency shown in Fig 3 top.) Interestingly, the tendency of this relationship persists even when the participant is induced to tilt slowly but substantially (Fig 4 top middle and right). This implies that the short-range high intrinsic stiffness manifests itself in a way that is independent of ankle torque and angle of the body. Stasis (low sway velocity) is its prerequisite.

Long-range stiffness is considerably less than short-range stiffness because it involves appreciable movement of the muscle tissues which now become much less stiff. Accordingly, the larger perturbations which measure long-range stiffness, or the movements associated with larger sway, destroy intrinsic stiffness and the correlation with sway velocity becomes lost. Because muscle stiffness is now comparable with connective tissue, altered muscle torque and thus muscle stiffness can materially alter long-range intrinsic ankle stiffness.

This explanation suggests that an individual’s connective tissue structure would determine their intrinsic stiffness. Because a high intrinsic stiffness can assist the nervous system to control equilibrium, sway velocity and personal stability would have an anatomical basis. The relevant connective tissues are the Achilles tendon and the structures of the foot. We were unable to estimate with our experimental setup the effect of foot compliance in our results. It is interesting that Gurfinkel *et al*. [45] showed a considerable range of foot compliance in different individuals. Unfortunately, they did not correlate the compliance of their participants’ feet with the size of their spontaneous sway. Loram and Lakie [12] showed that for very small stretches the compliance of the foot and the tendon was broadly comparable. Either structure might in principle set the upper limit of intrinsic stiffness.

This conclusion would have implications for pathology and therapy in several movement disorders. However, our results show only a correlation between individual short-range stiffness and individual sway velocity and cannot prove causality. An alternative explanation could be that the nervous system controls sway size and, by minimizing muscle movement, allows intrinsic stiffness to increase. On this basis, intrinsic stiffness would have a neural rather than anatomical basis. The correlation between stasis and high short-range stiffness is striking, but we cannot say whether the increased short-range stiffness causes the reduced sway or whether the reduced sway causes the increased short-range stiffness. We have previously measured intrinsic stiffness in conditions of zero sway (when participants were supported stationary in an inclined position) [19]. This could answer the causality question, but unfortunately, we did not do these measurements in the present experiments.

## Summary

In accordance with previous studies we show that there is a wide range of intrinsic ankle stiffness in different people and that some people spontaneously sway more than others. We show that in normal, minimally disturbed, standing there is an inverse correlation between the short-range intrinsic ankle stiffness of our participants and their spontaneous sway velocity. When intrinsic stiffness is measured with larger perturbations or while the participants are destabilized by tilting its magnitude is decreased and the correlation with stasis destroyed. Participants with higher baseline torque tend to have higher intrinsic stiffness.
